# Evaluation of delay in time to adjuvant chemotherapy after HIPEC and its impact on oncological outcome in advanced epithelial ovarian cancer

**DOI:** 10.1515/pp-2020-0103

**Published:** 2020-08-04

**Authors:** S.P. Somashekhar, Y. Ramya, K.R. Ashwin, S.Z. Shabber, V.K. Ahuja, R. Amit, K.C. Rohit

**Affiliations:** Surgical Oncology, Manipal Comprehensive Cancer Centre, Manipal Hospital, Bengaluru, India; Medical Oncology, Manipal Comprehensive Cancer Centre, Manipal Hospital, Bengaluru, India

**Keywords:** adjuvant chemotherapy, cytoreductive surgery, HIPEC, ovarian cancer

## Abstract

**Obejectives:**

Optimal cytoreductive surgery (CRS), followed by adjuvant chemotherapy, is a major predictor of oncological outcome in patients with advanced epithelial ovarian carcinoma (EOC). It is not clear if a delayed start of adjuvant chemotherapy negatively impacts on the oncological outcome.

**Methods:**

Prospective registry study on 75 patients treated with CRS and hyperthermic intraperitoneal chemotherapy (HIPEC). Adjuvant chemotherapy was started within 42 days in 41 patients (55%), later on in 34 patients (45%). Multivariate analyses of preoperative factors were done on survival outcome. Outcomes were recurrence-free survival (RFS) and overall survival (OS).

**Results:**

There was no difference in RFS after early introduction (median 35 months) vs. late introduction of chemotherapy (median 32 months), p = 0.17. Median OS in patients with late introduction of chemotherapy was 46 months and was not yet reached in early introduction group.

**Conclusions:**

In this exploratory study in a small group of women with advanced EOC, starting adjuvant chemotherapy more than 6 weeks after CRS and HIPEC did not deteriorate significantly RFS or OS. Well-designed clinical studies are still needed to evaluate the interplay of HIPEC and the point of time of postoperative adjuvant chemotherapy in this indication.

## Introduction

Annually, ovarian cancer accounts for 295,414 new cases and 184,799 deaths worldwide with age-standardized rate of 6.6 per 100,000 person-years [1]. Late presentation of the disease due to lack of effective screening methods, is a deterrent to favorable outcome. Standard treatment protocol entails a combination of optimal cytoreductive surgery (CRS) and platinum-taxane adjuvant chemotherapy. The interval between resection and chemotherapy provides an opportunity for micrometastases to proliferate. The kinetic changes following a surgical resection causes accelerated metastatic tumor growth due to shuttling of cells from G0 phase into the cell cycle [2]. Thus, delaying adjuvant chemotherapy has been proven to worsen outcomes of many cancers, including breast [3] and colon cancer [4]. While the optimal timing of chemotherapy remains ambiguous in epithelial ovarian cancer (EOC), it is suggested that increased time to chemotherapy may have a negative prognostic impact and thus delay beyond 4 weeks should be desisted [5]. However, in most large studies, after curative resection of ovarian cancer, adjuvant chemotherapy has been recommended only after complete recovery from surgery, which averages 42 days (6 weeks) [6], [7]. Feng et al. [8] concluded that a time interval up to 6 weeks between surgery and chemotherapy seemed to have no prognostic impact on patients with EOC. It has been reported that a longer interval between surgery and initiation of adjuvant chemotherapy led to a 22% decrease in OS of ovarian cancer while relative OS decreased by 4% for each week of delay in initiating adjuvant chemotherapy [9]. In a meta-analysis, Uson et al. [10] reported that time to adjuvant chemotherapy (between 20 and 40 days) following ovarian cancer surgery with curative intent was not associated with a higher risk of disease recurrence or death. However, this association was influenced by the rate of optimal debulking.

Ovarian cancer remains primarily localized to the peritoneum for most of its life and therefore intraperitoneal chemotherapy has been tried as a novel treatment option during first line adjuvant treatment in women with Stage III EOC [11] with better locoregional tissue penetration of chemotherapy drugs and reduced systemic toxicity [12]. Hyperthermic intraperitoneal chemotherapy (HIPEC) as a form of intraperitoneal chemotherapy, offers an added benefit of targeting the tumor cells with hyperthermia thereby enhancing the rate of necrosis and apoptosis [13]. Survival advantage of HIPEC in advanced EOC has been seen in a recent randomized control trial (RCT) [14]. Still, many clinicians are skeptical about HIPEC due to the fact that postoperative period might hinder the patient from receiving adjuvant therapies and thus the survival. However, neither the time to start adjuvant chemotherapy in patients receiving HIPEC, nor the effect of delay on oncological outcome has been defined so far.

This study aimed to evaluate the impact of the delay in starting adjuvant chemotherapy on survival outcomes in patients undergoing optimal CRS with HIPEC.

## Materials and methods

### Methodology

This was a prospective observational study conducted at the Manipal Comprehensive Cancer Centre, Bangalore, between February 2013 and December 2017. The study was approved by the Institutional Review Board/ Ethics Committee of Manipal Hospital (dated 23rd Jan 2013).

Patients with advanced EOC (Stage IIIc) having good performance status (Eastern cooperative oncology group – ECOG 0 or 1) with completion of cytoreduction score (CCR score) 0 or 1 were included in the study. Exclusion criteria entailed the exclusion of patients with non-epithelial ovarian cancer or with recurrent disease. Demographic factors like age, medical comorbidity (patient having one or more of any medical comorbidity like diabetes mellitus, hypertension, bronchial asthma, ischemic heart disease, deep vein thrombosis) were collected. Patients undergoing either upfront surgery or interval debulking surgery following neoadjuvant chemotherapy were included. Preoperative and intraoperative data were analyzed. All patients underwent optimal CRS with HIPEC. HIPEC was done by colesium technique at the end of CRS and was as per the institutional protocol [15] (Inj. cisplatin 100 mg/m^2^ over 90 min at 41.5 °C). Postoperative details including morbidity were mentioned. All patients received adjuvant chemotherapy on day-care basis after the recovery from surgery.

The primary goals were to evaluate the time to start adjuvant chemotherapy after the surgery and the effect of delay in time to start adjuvant chemotherapy in terms of treatment assignment. Time to adjuvant chemotherapy was recorded as the interval from the day of surgery to the first day of adjuvant chemotherapy. On the basis of available evidence in literature (large randomized controlled studies of adjuvant chemotherapy in ovarian cancer and based on a large retrospective study), an interval of more than 42 days (6 weeks) was considered as a delay in treatment [6], [7], [8].

The factors responsible for delay in adjuvant chemotherapy were analyzed. Mutivariate analyses of preoperative parameters were done on recurrence free survival (RFS) and overall survival (OS). The effect of delay in adjuvant chemotherapy was evaluated in terms of RFS and OS. RFS was defined as time from the surgery to first recurrence or death, whichever the earliest, OS defined as time from surgery to death of patient or last follow up.

### Statistics

Descriptive statistics such as the number (n), mean, median, standard deviation (SD), minimum and maximum were provided for all continuous variables. The duration of RFS and OS was estimated using the Kaplan–Meier Method. Mutivariate analyses of relevant parameters were done on RFS and OS. Paired *T*-test or Wilcoxon signed rank test was used to test the difference between RFS or OS based on the 42-day cut-off.

## Results

A total of 75 patients with advanced EOC (Stage IIIC) were included in the study: adjuvant chemotherapy was started within 42 days in 41 patients (55%), later on in 34 patients (45%). The demographic and baseline characteristics are presented in Table 1. Majority (86%) had high grade serous carcinoma ovary, with completion of cytoreduction (CC) score of 0 was 92% in whole cohort.

**Table 1:** Demographics and baseline characteristics of patients who had delay in chemotherapy and without delay.

	Without	With delay	Total	p-Value
	delay (n = 44)	(n = 31)	(n = 75)	
Age (years) (mean ± SD)	55.5 ± 9.9	54.9 ± 9.8	55.2 ± 9.8	0.79
Medical Comorbidity	20 (45.4%)	16 (51.6%)	36 (48%)	0.59
ECOG 0	38 (86.3%)	27 (87%)	65 (86.6%)	0.92
S.Hemoglobin (g/dl) (mean ± SD)	10.5 ± 1.16	10.27 ± 1.0	10.40 ± 1.09	0.36
S.Albumin (g/dl) (mean ± SD)	3.93 ± 0.35	3.95 ± 0.44	3.94 ± 0.39	0.75
Histological subtype				0.34
Serous carcinoma G3	35 (79.5%)	28 (90.3%)	63 (84%)	
Serous carcinoma G1	2 (4.5%)	2 (6.4%)	4 (5.3%)	
Clear cell carcinoma	3 (6.8%)	0	3 (4%)	
Mucinous carcinoma	3 (6.8%)	0	3 (4%)	
Endometroid carcinoma	1 (2.2%)	1 (3.2%)	2 (2.6%)	
PCI (mean ± SD)	10.0 ± 5.9	11.9 ± 7.3	10.8 ± 6.5	0.21
CC score 0	90.9%	93.5%	92%	0.82
1	9%	6.4%	8%	

The median interval of starting adjuvant chemotherapy in the overall study population was 41 days (range: 22–145 days) in the whole cohort. 45% of patients had a delay in starting adjuvant chemotherapy (>42 days interval).

Predictors of delay in starting adjuvant chemotherapy were presence of medical comorbidity, poor performance status, bowel resection, multivisceral resection, relaparotomy, postoperative morbidity. There were no difference in time to adjuvant chemotherapy between patients who underwent upfront or interval HIPEC (38.96 vs. 41.02 days, p = 0.95). Multivariate analysis of preoperative parameters (age, ECOG, CC score, medical comorbidities, PCI, upfront or interval HIPEC) on survival outcome showed no significant impact of these on RFS and OS.

**Figure 1:**
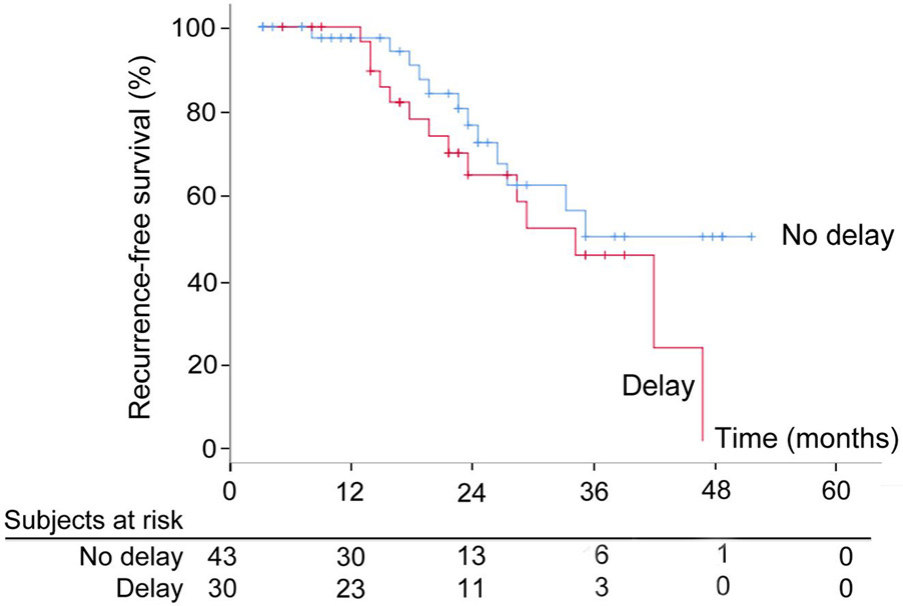
Recurrence free survival (RFS) of patients with (>42 days) and without delay (<42 days) in adjuvant chemotherapy.

**Figure 2:**
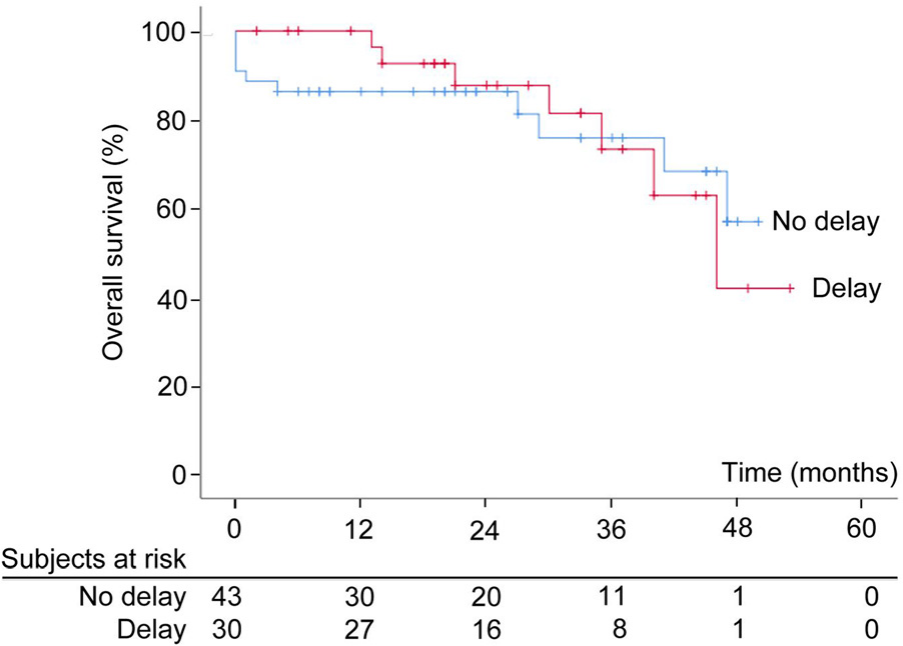
Overall survival of patients of patients with (>42 days) and without delay (<42 days) in adjuvant chemotherapy.

After a median follow up of 46months, the difference in median RFS was not statistically significant between the two groups with median RFS of 35 months as compared to 32 months in patients without or with delay in adjuvant chemotherapy (p = 0.17) (Figure 1). Median OS was 46 months in patients in delay group, however median OS not achieved in other group (Figure 2, Table 2).

**Table 2:** Recurrence free survival (RFS) and overall survival (OS) with or without delay in starting adjuvant chemotherapy.

	Median RFS, months	p-Value	Median OS (months)	p-Value
<42 days	35		Yet to achieve	
>42 days	32	0.17	46	0.82

## Discussion

Challenges persist in treatment of ovarian cancer especially as most cases are diagnosed at an advanced stage III. Areas of ambiguity remain in the optimal timing of chemotherapy following CRS and in the role of intraperitoneal chemotherapy. Various factors implicated in the delay in adjuvant chemotherapy are age, CCR, prolonged hospitalization and postoperative complications and these have predictive value [16], [17], [18]. In the present study, preoperative factors like medical comorbidity, poor performace status, intraop and postoperative factors such as bowel resection, multivisceral resection, postoperative morbidity, relaparotomy were associated with delay in adjuvant chemotherapy. However, neoadjuvant chemotherapy administration did not make any impact on time to adjuvant chemotherapy.

First introduced in the mid-1980s, intraperitoneal chemotherapy delivers a much higher concentration of drug directly to the peritoneal tumors in comparison to intravenous chemotherapy, thereby assuring a better safety profile whilst improving cytotoxicity [19]. A meta-analysis reported 10% increase in the number of patients receiving intraperitoneal chemotherapy leading to a 3.9-month increase in median survival time [20].

Studies have attempted to establish the prognostic relevance of the interval from surgery to initiation of chemotherapy [21], [22]. Established guidelines do not give any recommendation regarding the exact time interval to start adjuvant chemotherapy for ovarian carcinoma. Several studies have shown an improvement in OS with early initiation of adjuvant chemotherapy post CRS [5], [23]. A meta-analysis by Liu et al. [9] summarized the evidence from 15 cohort studies and deduced that early initiation of chemotherapy improved OS of patients with ovarian cancer. There was a decrease in relative OS by 4% for each week delay of initiating adjuvant chemotherapy. Tewari et al. [24] conducted a post-trial ad hoc analysis on ovarian cancer patients from a phase III randomized control trial and concluded that when time to chemotherapy exceeded 25 days, there was an increased risk of death in patients with Stage IV disease with complete resection. The studied interval between surgery and chemotherapy initiation has varied across studies. Most studies evaluated a 4–6 weeks delay, however Mahner et al., Flynn et al., and Feng et al. had cut-offs at 19, 22, and 15 days, respectively [8], [21], [23]. The present study was conducted to evaluate the implication of delayed adjuvant chemotherapy initiation on survival outcomes in patients with EOC undergoing optimal CRS. This study with Stage IIIC EOC patients had the cut-off of 42 days, which was based on the cut off used in other major studies [6], [7], [8]. 45% of whole cohort had a delay in initiation of adjuvant chemotherapy in patients undergoing HIPEC.

It was observed in this study that delay in chemotherapy, as defined by more than 42 days interval, had no significant impact on RFS in CRS + HIPEC group (35 vs. 32 months; p = 0.17).

During treatment with HIPEC high temperatures widen the sensitivity of cancer cells to the cytotoxicity of chemotherapeutic agents also leading to better penetration into the cancer tissues. A pooled analysis of nine comparative studies and 28 studies showed statistically significant survival advantage with HIPEC along with CRS and chemotherapy as compared to CRS and chemotherapy alone. Additionally, they noted that RFS was poorly reported in the comparative studies [25].

In the present explorative study, delay in adjuvant chemotherapy did not have significant impact on OS or on RFS. This may be attributed to the fact that a single dose of intraperitoneal chemotherapy administered at the time of surgery itself acted as adjuvant consolidation therapy.

Smaller numbers of patients with short term follow up were the limitations of our study. Statistical significance might have been reached with a larger cohort. There was no sample size calculation or power calculation beforehand for this study as this was not a randomized one.

The merit of our study lay in the fact that for the first time the association of delayed adjuvant chemotherapy and survival outcomes were compared in patients undergoing CRS + HIPEC. Being a single-center, a defined patient selection and established cytoreductive surgery protocol was executed.

## Conclusions

This study concludes that a delay in starting adjuvant chemotherapy didn’t have an impact in the HIPEC group owing to the fact that single dose of chemotherapy during surgery in heated environment may compensate for the delay. As this was an observational study, further well-designed clinical studies are required to evaluate the impact of single dose of intraperitoneal heated therapy and its interplay with the delay in starting adjuvant chemotherapy.
